# Webcams and Social Interaction During Online Classes: Identity Work, Presentation of Self, and Well-Being

**DOI:** 10.3389/fpsyg.2021.761427

**Published:** 2022-01-10

**Authors:** Alexandra Hosszu, Cosima Rughiniş, Răzvan Rughiniş, Daniel Rosner

**Affiliations:** ^1^Faculty of Sociology and Social Work, University of Bucharest, Bucharest, Romania; ^2^Faculty of Automatic Control and Computer Science, Politehnica University of Bucharest, Bucharest, Romania

**Keywords:** well-being, online education, webcam, presentation of self, identity work

## Abstract

The well-being of children and young people has been affected by the COVID-19 pandemic. The shift to online education disrupted daily rhythms, transformed learning opportunities, and redefined social connections with peers and teachers. We here present a qualitative content analysis of responses to open-ended questions in a large-scale survey of teachers and students in Romania. We explore how their well-being has been impacted by online education through (1) overflow effects of the sudden move to online classes; (2) identity work at the individual and group levels; and (3) Students’ and teachers’ presentations of self in the online environment, with a focus on problematic aspects of webcam use. The results indicate that both students and teachers experienced ambivalence and diverse changes in well-being, generated by the flexibility, burdens, and disruptions of school-from-home. The identities associated with the roles of teacher and student have been challenged and opened for re-negotiation. Novel patterns have emerged in teachers’ and Students’ identity work. Failure or success at the presentation of self in online situations is relevant for the emotional valence of learning encounters, impacting well-being. Online classes have brought about new ways to control one’s presentation of self while also eliminating previous tactics and resources. The controversy regarding webcams has captured this duality: for some, the home remained a backstage that could not be safely exposed; for others, the home became a convenient front stage for school. Well-being was affected by the success of individual and collective performances, and by student-teacher asymmetries. Overall, our study of online learning indicates powerful yet variable influences on subjective well-being, which are related to overflow effects, identity work, and presentation of self.

## Introduction

The COVID-19 pandemic has led to increased reliance on distance learning as a result of country-wide lockdowns and restrictions on in-person education (United Nations [UN], 2020). Among students and teachers, the effects of this abrupt shift have been ambivalent and diverse, with uneven combinations of positive and negative changes in well-being across different social categories. For example, the downturn in face-to-face instruction is expected to disproportionally affect teachers and students from disadvantaged communities, as well as those with previously poor education outcomes (Di Pietro et al., 2020). Significant segments of the student population became less engaged, or even lost touch with school, on a global scale, in countries such as Turkey (Kurt et al., 2021), New Zealand (Yates et al., 2021), Romania (World Vision, 2021), and the United States. This global effect was due to a combination of lack of internet connectivity and other household difficulties (Domina et al., 2021). Students often perceived online learning to be less interesting, enjoyable, and educational (Garris and Fleck, 2020; Loperfido and Burgess, 2020). Simultaneously, some students benefitted from the increased flexibility and reduced face-to-face contact that is part of distance learning (Burns et al., 2020; Garris and Fleck, 2020). Likewise, previous research finds ambivalence and heterogeneity among teachers. Some appreciated the flexibility, while others felt overburdened and isolated with increased stress, anxiety, and depression (Ozamiz-Etxebarria et al., 2021).

**FIGURE 1:**
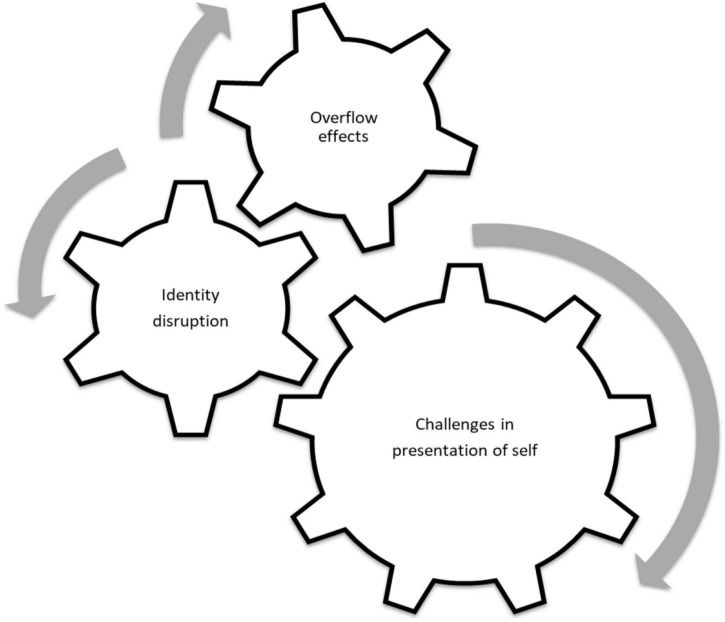
Three vectors of influence between online education and subjective well-being.

Our study is a qualitative content analysis of responses to open-ended questions in a large-scale survey of teachers and students in Romania. We examine how the well-being of students and teachers was changed by online education by focusing on three dimensions: (1) the overflow effects of online schooling on schedules, role conflicts, and other sources of positive and negative affect; (2) the re-negotiation of digital identity at both the individual and group levels through identity work (Schwalbe and Mason-Schrock, 1996); and (3) Students’ and teachers’ presentation of self (Goffman, 1956) in online environments, specifically addressing the webcam dilemma (Figure 1).

Subjective well-being is a concept that refers to individuals’ degree of satisfaction with their own lives, and it is typified by the predominance of a positive affect over a negative one (Diener, 1984). The turn toward online learning has shaped how teachers and students evaluate their lives, with important implications for their emotional states; and the impact has been multidimensional. *First, there have been overflow effects of the influence of online work and school on other spheres of daily life.* Heretofore private, teachers’ and Students’ homes have become public spaces, and their daily schedules have been modified to accommodate working from home, thereby radically changing traditional patterns of social interaction. There have been benefits from flexibility and saved time (for transportation to and from school, for preparing and dressing), but also costs from role conflicts and various additional requirements for distance learning (finding a place to study at home, problems with internet connectivity, and lack of appropriate devices, etc.). While certain people enjoyed withdrawing from public life and escaping constant scrutiny, this led to social isolation and feelings of loneliness in others (Burns et al., 2020; Villani et al., 2021). Moreover, the emergence of work-life conflict (Grünberg and Matei, 2019) varied with the presence or absence of young children in the family (Schieman et al., 2021), thereby leading to a heterogeneous reception of online teaching.

*Second, teachers and students have confronted challenges to their school-related identities.* Individuals create and recreate identities through social interaction. These identities are fluid, and people can change and adapt them to novel situations. Creating, maintaining, or changing identities requires individual and collective effort, which can be conceptualized as “identity work” (Schwalbe and Mason-Schrock, 1996). Individuals and groups develop signs (including labels, codes for interpretation, and rules of behavior) to indicate various features of the self. They use these signs to create images of themselves in interaction with others. Schwalbe and Mason-Schrock (1996) distinguish four distinct dimensions of identity work: determining group characteristics (defining), establishing the norms and rules of the group (coding), the existence of and/or creating occasions to express the identity of the group (affirming), and delimitation of those who belong to the group from the ones who do not (policing).

When we examine the shift to online education during the pandemic, it is apparent that the virtual environment stimulated *a reconstruction of identities* for both students and teachers. A recent study (Jones and Kessler, 2020) found that “the foundational pieces of teachers’ identities have been significantly altered, if not removed entirely, due to COVID-19” (p. 7), and this holds true for students as well. Increased responsibilities were imposed on students and their parents for planning learning activities, while their opportunities to request or receive advice from teachers decreased. Because of the sudden shift, numerous students “were not prepared to manage their learning in a space that provided them with less everyday interaction with their peers and their professors” (Loose and Ryan, 2020). In addition, moving assessment activities online has increased risks of students cheating (Bilen and Matros, 2021), thus heightening teachers’ suspicions and creating increased conflict between teachers and students.

Teachers also lost precious opportunities to connect and consult with peers, which affected their sense of collective identity and had a negative impact on their subjective well-being (Spicksley et al., 2021). They felt that the pandemic diminished their creativity, thereby reducing student engagement and exacerbating emerging forms of inequity and pandemic-related trauma among students, which degraded the teachers’ own emotional experiences (Loose and Ryan, 2020; Anderson et al., 2021). Occasionally, teachers managed to recover from the initial shock, while relying on relationships with peers and remaining deeply concerned about vulnerable students (Kim and Asbury, 2020). An ambivalent, yet overall negative, experience with the shift to online education is also reported by a survey of teachers and students in Slovenia: “The lack of daily commuting, improved eating habits, and more time available for a family were the main reported advantages of WFH. The main issues, highlighted by respondents, were higher stress levels, lower study/work efficiency, and poorer working environment at home. When comparing the online educational process with the traditional one, the absence of traditional laboratory work, inadequate social interactions, and limitations of online knowledge assessment were identified as drawbacks by both students and educators” (Bilen and Matros, 2021; Drašler et al., 2021).

Occasionally, the online experience was perceived as exhausting, becoming a source of negative effect. This feeling is referred to as “Zoom fatigue” (Johnson, 2020; Marquart, 2020; Smith, 2021). Bailenson (2021) identified four causes for this syndrome: (1) the eyes are being used unnaturally, as everyone is staring at each other for a large amount of time and people’s faces on screens are larger than in reality—“*you’re seeing their face at a size which simulates a personal space that you normally experience when you’re with somebody intimately”*; (2) the cognitive load is higher because of the work required to surmise the non-verbal messages of others; in real-life interactions, these flow naturally and without effort; (3) seeing our own faces all day long makes us more aware of ourselves and more critical of our appearances—“*Zoom users are seeing reflections of themselves at a frequency and duration that hasn’t been seen before in the history of media, and likely the history of people”*; and (4) there is a reduced ability to move and gesture during online activities, which negatively affects the creativity and efficiency of a meeting. Online interactions are perceived as artificial; even with cameras turned on, zoom fatigue is a problem for numerous individuals (Johnson, 2020; Mayer, 2020). Moreover, one might be distracted by one’s own face and the attempt to look good and interested, which tends to negatively affect focus: *“While looking at students may be comfortable for instructors, for students, it can be an alien experience”* (Finders and Muñoz, 2021).

*Third, the shift to online education implied that individuals had to suddenly present and defend their school-related identities exclusively in the virtual space, at the expense of face-to-face classroom situations*. The concept of the “presentation of self,” developed by Erving Goffman (1956), helps to elucidate how students and teachers managed their identities during online lessons. According to Goffman, individuals orient their actions depending on how they define the situation and how they would like others to perceive them. Both students and teachers were accustomed to curating their appearances in face-to-face educational interactions. However, online school required an adaptation of techniques as the “normal” was disrupted, and students and teachers both struggled to explore and exploit new ways of dealing with the virtual learning environment. Disrupted situations invite new registers of valuation, and combinations of old and new practices (Ciocănel, 2016, 2017). For example, the digital space offers the possibility of not being seen or heard, and the possibility of furtively moving attention to other digital activities, options that are unavailable in physical interactions. Shifts in the organization of the situation induce changes in the emotions experienced by interactants, who must cope with novel challenges in presenting their selves (Jderu, 2010). While Goffman (1956) theorized the presentation of self for face-to-face situations, the appearance of the internet made it increasingly relevant in digital spaces, particularly with the advent of social media (Pearson, 2009; Hogan, 2010; Bullingham and Vasconcelos, 2013). The context of massive digitization due to the COVID-19 pandemic and the sudden shift to distance learning have led to new arenas in which the presentation of self had to be tailored to cope with virtual situations (Flaherty and Rughiniş, 2021).

One of the foci of discontentment in online education during the COVID-19 pandemic has been the so-called “black boxes” phenomenon. This refers to the fact that students often prefer not to turn on their webcams during online classes (Castelli and Sarvary, 2020), despite teachers’ requests to turn them on for improved interaction and educational results: *“Speaking to a room of black boxes and profile images is quite debilitating and, frankly, weird”* (Cooper, 2020). Teachers have been making efforts to increase student engagement during online classes, but they continue to occasionally encounter black boxes (Pearson, 2020).

The digital leap stimulated a global debate, with educational experts, teachers, parents, students, and journalists putting forward arguments for and against compulsory use of webcams in online classes (Table 1). There are three main considerations concerning the use of webcams: (1) invasion of privacy; (2) digital inequity; (3) bullying, affected well-being, and increased anxiety.

**TABLE 1:** Opportunities and burdens of webcam usage during online education.

Opportunities	Burdens
1. Improved interaction, engagement, real-time feedback, and sense of community 2. Exercising control over students and monitoring their activity 3. Similarity with in-person classes	1. Social and digital inequalities that affect availability and how people are perceived by others 2. Invasion of privacy 3. Risks of bullying, anxiety, fatigue

In certain cases, students felt anxious when asked to turn on their cameras as they did not want their classmates to be part of their private space or did not feel comfortable seeing their own faces all the time (Reed, 2020). To combat this situation, some states and schools regulated the use of cameras during online education and established clear policies that state that cameras must be turned on for all students (Cooper, 2020). Reed (2020) argued that there are cultural differences from one home to another that could impact the ways in which students experience such policies. For example, girls who wear hijab in public spaces might not wear it at home, but would be required to do so if forced to turn their webcams on. Thus, teachers must not invade Students’ privacy: “*Allowing someone access to a live video of myself in my home is a privilege, not a right”* (Simoens, 2021). In addition, students have become more exposed to privacy and security risks. So-called “zoombombers” can infiltrate online classes and take personal information from the participants, occasionally even recording them (Moses, 2020) as not all schools have implemented privacy and security policies for online learning.

Digital inequity is another argument in favor of being flexible when it comes to camera policies in online classes. Students have varied socioeconomic situations and access to individual digital devices, efficient bandwidth, and space to study and attend online classes. Teachers must therefore question whether all students are able to use a webcam. In addition, some students have additional responsibilities, including housework or sibling carer activities, while attending online classes: *“It’s a privilege to be a student that can dedicate every waking moment to their studies”* (Nicandro et al., 2020).

Moreover, experts have expressed concern that forcing students to turn on their webcams might lead to increased stress and depression. Costa (2020) described how students and teachers might experience trauma from previous events and/or COVID-19 in a LinkedIn post: “*Our brains are doing exactly what they’re supposed to be doing during this crisis: focusing on survival.*” At the same time, bullying can occur when someone has their camera on and a classmate finds something funny or intriguing; it is possible that they record videos of the incident and distribute them to people outside the class.

On the other hand, teachers and educational experts advocate the benefits of turning on webcams during online classes, particularly with regard to student engagement (Will, 2020). There are three main arguments for having cameras on: (1) improved engagement and a feeling of community, (2) monitoring students, and (3) similarity with in-person classes.

First, engagement benefits derive from the immediate feedback received by teachers when they are able to see Students’ faces. Moreover, when students have their cameras turned on, they become more involved in the educational process as they attempt to present themselves as paying attention and doing the necessary work. Even though teachers are somewhere aware of Students’ reasons for not turning on their webcams, they cannot hide their frustration with the situation: *“I don’t want to guilt trip them and I know this is more about me than it is about them, but I can’t help but be annoyed that I’m teaching to a bunch of unanimated squares every day”* (Fagell, 2020). Teachers also expect that having cameras on during online classes would increase the sense of community and make the interaction more human, helping students feel less isolated and enjoying the company of their classmates (Rudolph, 2021).

Second, in the online environment, teachers find it difficult to supervise Students’ activity. Teachers describe situations in which they do not know whether the students were actually listening or had just logged on and disappeared. In turn, the students take advantage of their invisibility and find it easier to avoid answering questions, subsequently blaming their lack of participation on connectivity issues (Patrick, 2020). In this atmosphere of suspicion, teachers’ assumptions of student rationality and enlightened self-interest give way to diagnoses of student irrationality and self-deceit (Toth, 2013).

Third, teachers aim to create interaction patterns similar to in-person ones: *“Searching for the familiar is a common approach to coping with these uncertain times. Staring at an empty box or just a name seems to make many instructors uncomfortable, yet feeling such discomfort does not give them the right to demand entry into Students’ private spaces”* (Finders and Muñoz, 2021).

The Romanian approach to online education during the COVID-19 pandemic is similar to that of other countries. In March 2020, the Ministry of Education decided to close schools for a short period of time to control viral spread. During this first phase, the authorities did not institute clear regulations for transferring education to the online environment. The schools decided autonomously whether and how to proceed. By the end of April 2020, the government mandated online education, whether synchronous or asynchronous (Ministry of Education, 2020a) and, since then, the Romanian schools experienced various educational scenarios (online, face-to-face, hybrid) depending on the local and national pandemic situation. At the time of writing, because of the severity of the Covid-19 pandemic, in late October 2021, the Romanian Government has decided to close all schools for a 2-week vacation, rather than reinstate online learning. The Minister of Education has decried the strong inequalities of access that distort online schooling and argued that a vacation is preferable to reinforcing such inequity (Andronie, 2021).

## Materials and Methods

This research relies on an exploratory and qualitative case study. We analyze Romanian Students’ and teachers’ experiences with online interactions and webcam usage in primary (grades 0–8) and secondary (grades 9–12) school. Our study examines a subsample from an online survey conducted in November 2020. The questionnaire was disseminated by the NGO “Impreuna” Agency for Community Development, and the National Students’ Council in Romania, a country-level primary and secondary school Students’ union.

A total of 9,401 students and 3,265 teachers from all 42 Romanian counties answered the questionnaire. The study relied on availability sampling. The questionnaire included 8 closed questions that were similar for students and teachers, assessing how each category perceives online education (Annex – Questionnaire). Results indicated that students felt that teachers did not engage them sufficiently in learning activities, and they used collaborative teaching mechanisms less, compared to face-to-face education; they also believed that some teachers were not successful in adapting to the specificity of online education (Hosszu and Rughiniş, 2021). The quantitative data analysis also indicated that there was a dominantly negative, yet mixed, perception of online classes among students and teachers. With regard to students, 27% described online classes as “interactive” in a single choice question, but 17% described them as “relaxing,” 29% described them as “boring,” 17% described them as “useless,” and 10% selected the “other” option to mostly include negative attributes such as difficult, tiring, and stressful (Hosszu and Rughiniş, 2021, p. 4).

The current study focuses on a subsample of 5,372 students and 2,354 teachers who also nuanced their answers through the final open-ended question: “Please briefly describe your experience with online education.” The number of words used by the respondents varied between 1 and 707. We eliminated duplicate responses and those with fewer than 10 words, as longer responses were required for an in-depth analysis. Open questions in a survey have the role of eliciting participants’ stories in a less structured and limiting space, empowering the respondents to describe their own experiences. This dataset proved productive for identifying multiple and sometimes conflicting ways in which identity work and presentation of self-influence well-being, in online education.

The majority of students and teachers in our subsample are women from urban areas in Romania (Table 2). Even though the questionnaire was open for all pupils in primary and secondary school, most of the respondents in the student subsample (91%) were in high school (grades 9–12).

**TABLE 2:** Students and teachers’ subsample included in the analysis.

Subsample	Total	% Women	% Urban
Students	5,372	70%	86%
Teachers	2,354	93%	67%

We analyzed the findings based on three key concepts: overflow effects on well-being, identity work, and presentation of self. The units of analysis are the individual testimonials of students or teachers. We followed the six phase approach to content analysis articulated by Braun and Clarke (2006): (1) becoming familiar with the data by reading and re-reading the Students’ and teachers’ testimonies, (2) manually generating initial codes by finding patterns and differences in discourse within and between groups, (3) searching for relevant themes by iteratively identifying and grouping theoretically relevant codes, (4) reviewing emerging themes by excluding those that do not have enough evidence and combining those that are similar, (5) defining and naming resulting themes, and (6) writing the report. The thematic content analysis process was not linear, but reflexive and iterative. Peer debriefing through conference presentations, constant examination of data in light of previous studies, and reflexive documentation of codes and themes ensured trustworthiness at each step (Nowell et al., 2017). We developed the content analysis grid iteratively, by reading and re-reading open-ended answers related to the three concepts and identifying the relevant intersections (Table 3).

**TABLE 3:** Indicators used in content analysis of open-ended survey questions.

Category	Definition and examples	Dimensions	Indicators
Overflow effects on subjective well-being	Influences on individuals’ satisfaction with their own life and positive and negative effects (Diener, 1984) through changes in schedules, role conflicts, and requirements for work and learning intensity. Examples: “*I don’t have time to eat anything, I don’t have time to get up from the office; I sit for 6 h with my eyes on the screen, non-stop, in continuous stress*” (S1257). “*Me and the students feel tired, the eyes fill with tears, personal time no longer exists, we do not have adapted educational content*” (T543).	Positive evaluation	• Feeling safe and comfortable at home • Gaining the time previously spent in traffic or for preparations
		Negative evaluation	• Work/family role conflicts • Stress, anxiety, depression • Fatigue • Many hours spent in front of the screen (eye strain, back issues, sleep issues) • Pressure from supervisors (teachers, school principals, and official educational institutions) • Decreasing motivation and engagement • Missing in-person interaction
Identity work	Efforts made by individuals and groups to delineate, maintain, and adapt their identities (Schwalbe and Mason-Schrock, 1996). Examples: *“I am glad that some teachers are very involved and want to learn, they ask us for help” (S2981);* “*We learned that we are alone in this boat. The top of the hierarchy does not provide sustainable support for teachers*” (T103).	Defining and affirming	• Referring to group and/or individual • Defining the group • Missing the face-to-face, informal arenas that sustained diverse, nuanced identities (school breaks, extracurricular activities)
		Coding	• Strategies for adapting to online classes • Efforts for developing digital competences
		Policing	• Moral/informal sanctions • Blaming the other group • Unsatisfied with formal regulation
Presentation of self	Efforts made by individuals to present a convincing and convenient identity for an audience, and to receive validation from the audience, in a given situation (Goffman, 1956). Examples: “*I don’t feel well at all. I’m an anxious person and I don’t feel good in front of the camera. I feel watched and judged for my imperfections and other things*” (S2020) *“It is very unpleasant to teach in front of turned off webcams because you do not receive any feedback. In addition, many students stay in bed and may even fall asleep*” (T749)	Managing one’s online appearance	• Preoccupation with one’s looks • Preoccupation with how one’s environment appears to others • Gaining and maintaining control of the situation
		Managing webcam activity and demands	Preoccupation with: • Privacy risks • Gaining and maintaining autonomy and comfort • Quality of feedback • Quality of teaching and learning • Quality of assessment • Legal requirements • Relationships and communities

Taking into consideration the large dataset, we pursued an *a priori* thematic saturation (Saunders et al., 2018) related “to the degree to which identified codes or themes are exemplified in the data.” Thus, for each of the three concepts (Table 3) we searched for codes and themes in Students’ and teachers’ open-ended answers, until we ceased finding significantly novel elements. We read the answers repeatedly, re-examining their significance for our research aims. The data proved to be rich in mapping personal experiences over a certain code or theme and, as Corbin and Strauss (2015) pointed out, the researcher can always “add new properties and dimensions to categories.” This is also why we used multiple testimonies to illustrate the diverse patterns in discourse for both students and teachers. Students’ and teachers’ testimonials were coded based on their position in the database—for students the codes are between S1 and S5372, while for teachers the codes are between T1 and T2354.

We benefitted from having diverse backgrounds in our co-author team, combining teaching experiences in social sciences, in engineering, and in entrepreneurship education. This form of researcher triangulation (Denzin, 2009) helped us better capture the ambivalence of technologically mediated interactions, which can feel both empowering and depleting, at the same time.

The testimonies were coded by two of the authors, each rating independently all answers. Based on a random sample of 100 answers (testimonies) for both students and teachers, the proportion of overall agreement between the two raters was 83% with a Kappa value of 0.83, indicating a strong interrater reliability.

## Results

### Overflow Effects of Online Classes on the Well-Being of Students and Teachers

To obtain an overview of dominant topics and affective reactions to online classes, we counted the number of times keywords related to well-being were repeated in Students’ and teachers’ testimonials (Table 4). We applied a Chi-Square Goodness of Fit Test to assess if keywords differ significantly between students and teachers. For 8 out of 10 words there is a significant difference (bolded in Table 4). Only for “concentration” and for “test” the *p*-values are higher than 0.05, indicating that the two keywords are used in comparable proportions by students and teachers. The most frequently used word by students in describing their experience with online education was “tired” or other derivates of the word (866 times). Occasionally, fatigue was accompanied by stress, anxiety, frustration, or depression. Some students described the online lessons as boring (284 appearances), while the teachers referred to the students as being bored during online activities. When analyzing the frequency of words used, the students were more preoccupied by the webcam debate compared to the teachers. Students also referred more to “stress,” “fatigue,” “anxiety,” “bored/boredom,” and “grades.” One of the words that teachers used more frequently than students was “learning” or its derivates, as the teachers invoked their personal learning process in the online context and described how they adapted to the new educational format. Teachers also invoked “adaptation” significantly more often than students.

**TABLE 4:** Keywords repeated in students’ and teachers’ testimonials.

Keywords related to well-being	No. of times the word appears in Students’ testimonials (*n* = 5,372)	No. of times the word appears in teachers’ testimonials (*n* = 2,354)	Chi-Square (Sig.)
Stress	**293 (6%)**	20 (1%)	89.28 (0.00)
Fatigue	**866 (16%)**	95 (4%)	219.48 (0.00)
Concentration	169 (3%)	80 (3%)	0.34 (0.56)
Anxiety	**59 (1%)**	1 (0%)	23.68 (0.00)
Bored/boring	**284 (5%)**	16 (1%)	93.01 (0.00)
Camera/webcams	**306 (6%)**	52 (2%)	45.04 (0.00)
Test	144 (3%)	70 (3%)	0.52 (0.47)
Grades	**164 (3%)**	39 (2%)	12.47 (0.00)
Learning	338 (6%)	**317 (14%)**	108.58 (0.00)
Adaptation	192 (4%)	**335 (14%)**	292.47 (0.00)

*Significant values are highlighted in bold.*

Through qualitative content analysis, we identify two perspectives on Students’ changes in well-being, as described by the students themselves. On one hand, some said that online education made them feel less stressed than previously. These students felt safe at home, were comfortable with their space and family, and some even had additional time for hobbies or preparing for national exams: *“I can say that I feel quite safe considering that I am in my room. I like that I can wake up later and dress much lighter,”* (S4324, female, 10th grade) or “*Sitting at home, I find comfort and I can say that I do not dislike this, because it does not distract me from learning, on the contrary, it is easier for me to be careful and work correctly*” (S4688, female, 9th grade).

On the other hand, other students experienced online education as marred by anxiety. Fear regarding COVID-19 was coupled with familial anxiety arising from parental unemployment or other financial problems. The shift to online schooling involved additional layers of stress: (1) there was more homework from teachers, and students felt unprepared, (2) students had to spend many hours in front of screens both for online classes and for homework: “*I don’t have time to eat anything, I don’t have time to get up from the office; I sit for six hours with my eyes on the screen, non-stop, in continuous stress”* (S1257, female, 11th grade), and (3) students also received more or different forms of criticism and pressure from teachers: *“I have a feeling of increased anxiety lately due to threats of low grades, evaluations, and tests, or being counted as absent due to a video camera not being turned on. I feel as if the mental and emotional health of the students is much more neglected than it was before”* (S1257, female, 11th grade). These feelings were aggravated when students did not possess appropriate digital devices and space for study, or when students had additional household duties. Students also witnessed an increased pressure from teachers for subjects that were usually considered “less important” in face-to-face activities and had not previously been a cause for concern (such as Arts, Music, Sports etc.). Students noticed that certain teachers became more conscientious, as they were being more closely monitored by school management, by the parents who could hear the lessons, and by other possible spectators who had access to a recording from the lesson.

*I understand that you are monitored, but to start learning music when we had previously studied completely different subjects or played different games during music hours is quite unnecessary. Making us jump around the house during Sports and be on the webcam, so as not to be marked absent is even more unnecessary (S1390, female, 10th grade).*

Furthermore, some students perceived as unfair the sanctions they received for being late, having issues with internet connectivity, or for dealing with private matters during the online classes: “*There are also teachers who shout at you when you need to do something at home and you are late for classes*” (S2475, female, 10th grade). For students, teachers’ reactions appear as self-serving, following a double standard: “*When they have a problem, it’s normal, and when we have a problem (the internet can’t cope, or it’s hard for me to work on the phone and turn on my microphone) we are ‘terrible’*” (S3975, female, 11th grade). The students expected teachers to be more empathetic: “*We should be a team, we should not blame them for anything, and they should not behave as if everything is the same*” (S4167, female, 10th grade) or “*it is very frustrating and annoying, especially when they ask us to be patient. We must be patient with them, but why does no one have patience with us?*” (S3918, female, 10th grade).

In certain cases, Students’ motivation decreased: “*The well-being decreases, the motivation no longer exists and the classes are more and more tiring, more inefficient. The number of absences is increasing for various reasons. Information cannot be retained as easily and probably in many cases all these obstacles and feelings end in severe depression*” (S3642, female, 11th grade).

The teachers also mentioned that their well-being was affected by both the changes to learning and education and the pandemic in general. They described feeling tired and unmotivated: “*Me and the students feel tired, the eyes fill with tears, personal time no longer exists, we do not have adapted educational content*” (T543, female). “*It’s getting harder and harder, we (teachers, parents, student) are more and more demotivated*” (T274, female). Moreover, a few teachers were unable to see the potentially positive outcomes of their struggles (Table 5).

**TABLE 5:** Overflow effects on the well-being for students and teachers.

Dimension of well-being	Opportunities for positive affect	Burdens for positive affect
Students	Feeling safe and more relaxed at home Enjoying time spent with family Autonomy in organizing their time	Anxiety Boredom Social isolation: decreased extracurricular interaction with peers Many hours spent in front of screens Lack of access to digital technologies deriving from digital inequalities Sanctions and conflicts with teachers
Teachers	Personal development: Rising to the occasion by enhancing their digital skills and creating new teaching methods	Fatigue Role conflicts generated by the superposition of working and home time and space, and by multiplication of work tasks Lack of access to digital technologies deriving from digital inequalities

### Students and Teachers’ Identity Work in Online Education

The new online learning environment has incited students and teachers to adjust their identities and reconstruct their social interactions, thus protecting or enhancing their own well-being. In order to explore how students and teachers re-created their online identities, we used Schwalbe and Mason-Schrock’s (1996) concept of identity work and its dimensions. We grouped “defining” and “affirming,” because we witnessed respondents’ identity definitions in their attempts to affirm them, in the open survey answers. We also identified new forms of “coding” and “policing”.

With regard to *defining and affirming* their identities, students usually referred to themselves using the plural pronoun “we” and defined themselves as in opposition to the other group-teachers. Most students expressed some criticism of their teachers in the survey. These criticisms included the following aspects: (1) they (teachers) are not well prepared for online teaching as they have limited digital skills—“*Teachers should have been trained by the Ministry so that they knew how to use online platforms*” (S13, male, 11th grade); (2) they (teachers) do not respect the rules and ask students to turn on their webcams, or even buy a webcam if they do not own one—“*Even if we say that it is not mandatory to turn on webcams, no one listens to us*” (S720, female, 11th grade); (3) they (teachers) give too much homework and believe students have more free time because they are at home; (4) they (teachers) are very suspicious and believe that students are cheating or lying—*;* “*If they hear a sound in our room then they immediately think that we are cheating and give us a 4 (grade)*” (S483, female, 10th grade). Students also felt that their voice did not matter and that no one really cared about their experiences: “*I am a student and, even if my voice were heard, no one will take it seriously, no one takes an interest in us, we know this*” (S4266, male, 10th grade).

Therefore, students *affirmed* their shared identity as a group using two layers of complaining techniques: (1) general complaints regarding the negative effects of online education (they are anxious, tired, unmotivated, etc., as discussed previously), and (2) complaints regarding teachers’ inability to adapt constructively to online education, relying on threats and sanctions rather than positive motivation.

The students *coded* their identity into social norms and strategies to adapt to the online classes. One of these norms was to adjust timetables and dressing codes, relying on the flexibility of home etiquette: “*the good part is that, from home, we can eat more consistently during breaks than we did at school and we can get dressed in classes the way we want*” (S4140, female, 9th grade). Thus, most of the students mentioned that they enjoyed waking up later than before, because they did not have to prepare for or commute to school. Some preferred participating in online classes from their beds, while others did so because they did not have the opportunity to use a desk or an adequate chair. Moreover, while eating during classes was either forbidden or discouraged in face-to-face school, some students revised this norm and appreciated the ability to eat during class time, without being seen by others. Even so, all of these norms of acceptable comfort were challenged and even eliminated if the students had to turn on their webcams.

Students also devised norms to protect their image and private space. They attempted to defend their right to decide whether or not to turn on the webcam and considered that it was stipulated by Romanian regulations^1^ *“I argue with all the teachers because they cannot understand a law given by the state and forced me to buy a webcam”* (S2167, male, 12th grade). In addition, the students mostly avoided turning on their webcams if it was not in fact enforced by teachers; thus, it became the informal norm to have them off: “*And if the law says that students do NOT have the obligation to keep the webcam on, teachers should understand that as well*” (S3965, female, 9th grade).

Furthermore, students *policed* their collective identity by formulating it in contrast and conflict with teachers: “*It irritates me that, sometimes, when some of my colleagues don’t even have internet connection, electricity or the possibility to turn on the webcam, the teachers are intolerant, they make us liars, saying that these are just excuses for not attending classes*” (S264, female, 12th grade). Consequently, they also attempted to create some form of sanctions, whether formal or informal, for the teachers: “*A change could come also from the teachers, because if they would not act as if they were superior and as if we were robots, maybe our respect for them would come naturally and we would learn even in a time like this*” (S4583, female, 10th grade). At the group level, the decision to not turn on webcams acquired the secondary significance of a method for punishing teachers. Other forms of sanctions included limited participation and displaying a lack of motivation to learn: “*We are sometimes punished if we do not turn on the camera, which sometimes makes us act against the teacher and pay less attention*” (S2457, female, 9th grade). In contrast to Students’ collective voice, the teachers usually *defined* their experiences with online education at an individual level, as they mostly felt alone in the process of adapting to online teaching. Teachers were affected by the reduction of meaningful interaction with their peers and students: *“I miss the physical presence of my students; I miss communicating with them and with my colleagues; I miss my Students’ smiles”* (T293, female). Thus, teachers’ well-being was influenced by the Students’ refusal to turn on their webcams; indicating that it made them feel as though they were talking to no one and kept them from receiving feedback in real time: *“It’s sad to talk to a class of students without seeing them”* (T12, female). Regardless, teachers expressed satisfaction with their personal progress in terms of their digital skills and capacities to teach online—“*I improved my digital skills both by participating in courses and individually; I discovered and created various materials that I use in online teaching*” (T2191, female).

The social norms and practices *coded* during online teaching varied between teachers (“*Many educational materials created by me, a lot of visual support and attention-grabbing/holding exercises*” (T26, female); “*I learn together with my students, we support each other in the instructive-educational process, discussions and debates, videos, worksheets on various platforms*” (T1213, female). The teachers constantly expressed concern for students who could not attend online classes due to a lack of access to devices, limited internet connectivity, lack of an appropriate space for learning, or other duties in the home. One of the norms the teachers adopted involved supporting these children in obtaining tablets or internet connections or giving them materials and exercises that could be done on paper without attending classes. The teachers were preoccupied with finding innovative teaching methods in order to help the students feel motivated. Even though some of the teachers were satisfied with the feedback they received from the students, others complained about their lack of response.

*I found that the Students’ motivation decreased despite the fact that I tried hard to come up with all kinds of interactive materials and make them feel less apathetic. They just want to be left alone. They don’t turn on their cameras, I don’t see them, I literally don’t know what they are doing behind the monitor. I have no way of knowing if any of them need more support or if they are going through a difficult period. Sometimes I feel like I am talking to the wall (T1139, female).*

With regard to *policing*, teachers affirmed their group identity in relation to two categories of actors: (1) students and parents, who were portrayed as detached and uninvolved; and (2) the local, county, and national administration, who were perceived as making schooling more difficult through excessive bureaucracy and confusion. In several instances, teachers expressed the fact that “good” students continued to study, actively participate in classes, and do their homework, while the “not so good” students increasingly neglected the educational process, taking advantage of being unseen and uncontrolled during online classes: *“I believe that the only competence developed by online education is that of a spectator. Cameras off, microphones off…. Total indifference”* (T13, female).

In other cases, the fact that students and teachers experienced issues with online education became an opportunity for them to *support* each other in identifying strategies for adaptation. For example, students helped teachers use their digital devices and specific online platforms, while teachers who were concerned about their Students’ emotional health, talked to them about their feelings: “*I am glad that some teachers are very involved and want to learn, they ask us for help*” (S2981, female, 11th grade); “*some teachers ask us for our opinion and we come up with suggestions for how to conduct our classes and it is very ok when that happens because they and we still discover the ‘right way’ to organize ourselves*” (S199, female, 9th grade) (Tables 5, 6).

**TABLE 6:** Students’ identity work—opportunities and burdens.

Students’ identity work	Opportunities for maintaining and developing school-related identities	Burdens for maintaining and developing school-related identities
Defining and affirming identities	Formulating a collective “We, the Students” General complaints about negative effects of online education Complaints about teachers’ inability to adapt to online education	Anxiety Boredom Exhaustion Loss of peer-to-peer and informal social spaces: school breaks, free times before and after class
Coding new norms and symbols	Embracing the advantages of online education: flexibility of the household etiquette, waking up later, comfortable dress code Protecting their image and private space	Too much homework and too many assessments The emerging norms of online education were limited when the turned-on webcam was mandatory
Policing ingroup vs. the outgroup	Creating a group identity in opposition to teachers, seen as the “other group”	Moral/informal sanctions for the teachers Blaming teachers as the “other group”

Regarding their relationship with the state, teachers mostly perceived that the Ministry of Education was not meaningfully involved: “*The ministry/government does not give a damn about the situation on the spot and only sends punishments and request after request. Everyone’s well-being is no longer important, only threats and fear. It would be good for them to treat us and the children as human beings*” (T1059, female). In addition, the teachers believed the Ministry and other entities overloaded them with bureaucratic tasks and were obsessed with teachers proving that they were working: “*It is much harder to prepare for these classes, and the many requirements of the Ministry of Education and the stress they cause me take away from the time I could be using to prepare more interesting materials for children. I personally work more than 10 hours a day for school. My family is neglected*” (T1016, female); “*There are health problems caused by sitting for over 6 hours a day in front of the computer + mental exhaustion + destruction of eyes + radiation of the whole body + lack of time to solve personal/family problems!*” (T2193, female). Some teachers expressed an increased mistrust toward the higher administration: “*The lessons learned during this pandemic situation were to no longer trust those who lead us (…) and to manage everything alone*” (T1431, female). They felt that the responsibility for facing challenges fell on them alone: “*I understand the need for online education in these moments, but they put everything in our hands! We were neither trained nor prepared for such a deployment of forces*” (T21, female).

Online education removes precious arenas for Students’ identity work, including breaks and free time before and after class. These intervals allowed students to present and develop multiple, nuanced facets of their identity in relation to one another, as peers, and in relation to their teachers. With the restrictions imposed by online school, these social spaces are lost: “*you spend a lot of time socializing ‘face to face,’ which is impossible in the online environment*” (S1207, male, 11th grade); “*there are some aspects of physical presence at school that no matter how dedicated, prepared and benevolent teachers may be, they cannot fulfill it through online education*” (S3570, female, 9th grade). Students decry the resulting monotony and emotional void: “*We were doing stupid things, but, at least, we had fun*” (S4559, female, 6th grade).

The online design creates multiple barriers, and some pupils felt as strangers, outsiders or robots: “*I don’t feel like I’m in class, I miss my colleagues, my teachers, it’s like I’m in front of a wall, the screen doesn’t transmit my emotion*” (S2333, female, 9th grade); “*It is difficult for us, being between four walls, alone. Sometimes it seems like the world is standing still. It’s empty, deserted, sad…”* (S1096, female, 9th grade); “*I don’t feel like I belong to a class*” (S3818, female, 12th grade). Some of the teachers considered, as well, that online education is somehow useful, but limited in ensuring the flows of social interaction that students and teachers need to relate to one another meaningfully: “*The day-to-day meeting of the teachers with the students outlines the character and develops their personality. Online education is a farce*” (T1254, male), “*Online learning cannot be compared to face-to-face learning*” (T1160, female).

### Students’ and Teachers’ Presentation of Self in Online Classes: The Webcam Dilemma

In face-to-face educational activities, one cannot participate without one’s entire body being present in the classroom, but participation changes meaning in online classes. The students consider participation to mean being connected to the online meeting, without necessarily turning on their webcams or microphones: *“I believe that you can show interest in the classes even if you have your webcam turned off”* (S1767, female, 9th grade); “*if they can’t see me, that doesn’t mean I’m not paying attention*” (S4501, female, 9th grade); “*I can learn without seeing the teachers*” (S2487, female, 9th grade).

However, teachers complain about the scarce immediate feedback from students (through non-verbal gestures), as they felt unable to evaluate whether the students needed more clarification in real time (“*I don’t have visual feedback, I’m not convinced that they understood”* (T128, female); “*I teach in front of a black screen. So, zero feedback*” (T368, female); “*The Students’ assessment is unsatisfactory given that most of them do not turn on the webcams and you cannot see the quick feedback of your actions*” (T2171, male); “*Unfortunately, fewer and fewer students appreciate these efforts. It is very unpleasant to teach in front of turned off webcams because you do not receive any feedback. In addition, many students stay in bed and may even fall asleep*” (T749, female) (see also Castelli and Sarvary, 2020, p. 3566). Teachers drew their energy from their interactions with the students, and without it, they felt *“alienated”* or “*like strangers”* teaching in a non-human, unreal context—“*I miss my children through their physical presence, I miss the communication relationships with the children, with my colleagues, I miss the smiles of my students*” (T293, female) (Table 7).

**TABLE 7:** Teachers’ identity work—opportunities and burdens.

Teachers’ identity work	Opportunities for maintaining and developing school-related identities	Burdens for maintaining and developing school-related identities
Defining and affirming identities	Care for students Autonomy in organizing classes Enhancing their digital skills	Scarce opportunities for sharing their individual experiences Feeling alone in the process Neglecting their personal and family priorities
Coding new norms and symbols	Reorganization of teaching, homework, and assessments Receiving help from the students	Difficulties in developing digital skills and teaching methods
Policing ingroup vs. the outgroup	More focused on Students’ potential and results Engaged in finding solutions for disadvantaged students	Unsatisfied with the formal regulations from local and central authorities Complaints about Students’ and parents’ detached attitude

Seeing what the students were doing during online classes gave the teachers the impression of having some form of control over the students and their educational achievements: “*We have extremely low control over Students’ activity, especially when webcams are turned off*,” (T1211, female); “*we have no control over the students, they are distracted from activities, they are not fully involved!*” (T20, female). The webcam debate also involves trust and the lack thereof. Teachers did not trust the students to own their educational process behind blank screens and instead suspected them of not paying attention or engaging in other activities. Occasionally, the students appeared to confirm these expectations, from teachers’ perspectives, by insisting on not turning on their webcams. On the other side of the digital barricade, interpretations differed and reactivity influenced behavior. Even the students who did not mind using their cameras said they decided not to do it because of the teachers’ obsession with sanctioning them if they were turned off: “*In general, I voluntarily turn on my webcam because it seems like common sense, but when it is made obligatory, I become reluctant, develop an issue with the teacher, and lose interest in the class*” (S3089, female, 11th grade).

Furthermore, due to teachers’ mistrust, the assessments and grading in the online learning environment became a challenge for both parties. The teachers attempted to identify secure evaluation methods, to ensure that students would not cheat. For example, the students complained about 10-min tests that are impossible to be solved, while teachers limited the time with the aim of diminishing the possibilities of cheating: “*Scoring is unfair. We receive artificial grades (maybe even lower than we deserve) or we have unfeasible tests in terms of time (4 minutes for 10 questions of medium to high difficulty, not having time to read all the questions)*” (S3603, female, 11th grade). Students also denounced situations where teachers asked the students to close their eyes and put their hands behind their backs to ensure students were not using additional materials to answer questions: “*torturing students with listening with their hands behind their backs is not a solution, I believe that we have passed the communist era for some years and we should be evaluated differently, through teamwork projects and not encouraging ‘grades hunters”’* (S3183, female, 10th grade). A few of the students believed that teachers need to focus on innovative teaching methods rather than expecting the students to reproduce the lesson verbatim: “*If the student wants to cheat, he will, most likely, find solutions and it is useless to waste your energy catching students who have not learned instead of focusing on teaching in a way that is as interactive and practical as possible*” (S2563, female, 11th grade). The grading and cheating issue is not new, but it became a more stringent aspect in online education, and it is linked with the teaching methods used therein. According to the students, the teachers should have identified more innovative teaching and grading methods and given up the memory tests with an increased focus on debates, team projects, and interactive evaluation: “*The Romanian school has not evolved at all in the last 20 years. The big problem of Romanian education is the mentality of teachers, who do not value the Students’ knowledge, but their ability to memorize information*” (S3965, female, 9th grade). On the other hand, the teachers found it very difficult to assess the Students’ learning progress in an online environment: “*The evaluation is almost impossible to do in the classic way. Online tests are very easy to fool. A student in the class solves the test and sends them to everyone in the class*” (T148, male). The teachers mentioned they had fewer possibilities for sanctioning the students who did not learn because the school administration and the Ministry of Education promoted the idea of being more indulgent with the students due to the difficult times they were undergoing: “*The trend is toward hyper protection for the students, not to give them (anymore)*” (T1988, male). According to the teachers’ accounts, the parents also participate in their children presentation of scholarly selves by, for example, whispering the correct answers into the children’s ears—“*Unfortunately, some parents intervene and whisper answers to students, believing that this helps them*” (T89, female); “*the only thing that bothers me in teaching online is the fact that some parents do not understand to sit at the back and not to whisper*” (T166, female).

Some of the students believed that turning on their cameras would allow others (classmates and teachers) into their private space, which would contribute to enhanced anxiety: “*I don’t feel comfortable at all; I’m forced to turn on the webcam when it’s not necessary to do so. I’m stressed all the time; I can’t concentrate at all*,” (S779, female, 11th grade); “*I don’t feel well at all. I’m an anxious person and I don’t feel good in front of the camera. I feel watched and judged for my imperfections and other things*” (S2020, male, 10th grade). This concern was fueled by international reports (UNICEF, 2020) and personal experiences of students who were bullied by their classmates, who took pictures of them and then distributed them on different social media channels: “*Many students do not turn on their webcams because they are afraid that some of their classmates will take screenshots of them, and unfortunately, even if it is illegal, this often happens*,” (S3878, female, 10th grade); “*the presence of other people during the lessons is a disturbing and dangerous factor, because some students may be ridiculed for their behavior or opinions*” (T89, female).

The students felt that the rest of the class was staring at their walls or at some other person behind them: “*I will never understand this necessity of opening the camera; a student can pay attention and be involved even without the teacher seeing his walls, his little brother coming into the room, or the cat walking on the desk*” (S4, female, 11th grade). When the webcams were on, the students could not control their appearances and could be labeled and judged by the spectators (students and teachers): “*All I feel is a constant fear that I will do something wrong or do something that will make the others laugh at me”* (S4501, female, 9th grade). One of the students said that she wanted to go back to school where *“my mother does not enter and call me to eat and my brother does not sleep in the same room*” (S1574, male, 9th grade).

Furthermore, the students were occasionally reprimanded by the teachers for the noise in their rooms, eating, or someone else talking in the room. There is some dissonance regarding teachers’ expectations from the students in this regard: teachers wanted students to turn on their webcams, but they did not want the lesson to be disturbed by what was happening in Students’ houses. The interactional frictions of online learning meant that the social distance between students and teachers often increased due to the lack of trust and mutual understanding (Table 8).

**TABLE 8:** Students and teachers’ presentation of self and the webcam dilemma.

Presentation of self and the webcam dilemma	Opportunities for a competent presentation of self	Burdens for a competent presentation of self
Students	Autonomy in controlling their presentation of self by restricting general visibility, the information that they give off involuntarily and the intrusion of backstage elements in the front stage of school interaction	Overlapping of public and private stages of interaction Lack of an appropriate, isolated space for learning Professors’ suspicions of dishonest behavior and coercive strategies The risk of being bullied by colleagues and intruders
Teachers	New teaching and assessment methods	Lack of control over the classroom Lack of feedback from the students Degradation of the emotional richness of interacting with students; feeling alone and abandoned by students Suspicion of Students’ dishonesty or disengagement

The teachers also feared their private lives appearing online, but, at least in our survey data, they mentioned it less than the students: “*I have difficulties due to the lack of a high-performance computer/laptop on which to design my lessons for my students, I am a single mother with 2 children, I did not receive any help, space is also a problem, I pay rent. I don’t have time to help my children, the workload is very high*” (T2114, female); “*I lock myself in a bedroom while the grandmother stays with the children, but there are background noises and small disturbances*” (T63, female). Further, there were situations in which teachers felt the intrusion of the school management during online classes: “*Laws are given, but interpreted at the discretion of the school principal who enters abusively, without prior notice at the online classes. It is chaos and only creates inconvenience!*” (T1063, female); “*For teachers no one did anything! Instead, now, the school principals and / or inspectors have started to enter our online classes on the grounds that they are ‘monitoring us’!*” (T1830, female).

## Discussion

The sudden shift to online learning has disrupted the typical interaction situations in face-to-face education. The resulting anomie led to friction, relaxation, and innovation for both students and teachers. The impact on well-being has thus been heterogeneous and affected by systematic sources of inequality. Our results converge with previous research, documenting overflow effects from working and studying at home, disruption of student and teacher identities, and changes in tactics and resources available for a successful presentation of self. Our study also highlights the importance of collective identity work and the role of student-teacher asymmetries.

The teacher/student relationship is defined by both collaboration and subordination, by converging but also diverging interests, thereby resulting in a combination of supporting and conflicting interactions. *The sudden transition to digital settings has upended the relatively stable, established patterns of collaboration and adversity created in face-to-face education situations*. According to our analysis, professors and students have both lost and gained in terms of their methods of influencing their counterparts. The identities associated with the roles of professor and student have become open for re-negotiation and re-definition, with novel patterns of individualization and relationships, alliances, and community-building.

The presentation of self in online learning situations was a specific focus of this paper because of its relevance to the emotional valence of learning encounters. *Online classes have brought about new means of controlling one’s presentation of self and to express oneself, while also disrupting previously employed tactics and resources*. In any interaction, participants make efforts to pursue a favorable definition of the situation and avoid interactional trouble, such as being embarrassed or giving out information that conflicts with their desired identity. Interactional trouble can be very stressful for participants, and the individual ability to control one’s presentation of oneself and to handle negative events is highly variable. Consequently, there has been a systematic increase in uncertainty and in opportunities for both success and failure in controlling presentations of self. For some individuals, this change has amplified anxiety, fatigue, and stress, all of which have negative consequences on overall well-being. Conversely, others have experienced this transformation as an increase in autonomy, social relaxation, and time flexibility, leading to an enhancement in well-being. However, there are mixed scenarios as well. Personality traits and contextual factor, including the human, family, community, and technological resources available to students and teacher, have made the difference in their ability to cope with this disruption and transform it into a positive, rather than negative, subjective experience.

Examining identity work and the presentation of self also reveals how the impact of online learning was very much a *collective* result. Individual experiences depend on the joint, coordinated *classroom* performance when it comes to re-defining identities and managing failures and deviance. The *household* of an individual (whether teacher or student) also plays an important role in making room and time for online learning, which leads to either a stressful or a favorable learning and teaching atmosphere.

For certain people, the home remained a backstage, which could not be exposed without risking humiliation, while for others the home became a convenient front for learning performances, offering more comfort and reduced time costs than in-person school. There is an *asymmetry*: *students appreciated the freedom of low visibility more than teachers*, who systematically decried the lack of feedback and human connection. Still, *both students and teachers resented the alienation* brought about by the reduction of interactions to formal, classroom activities, as breaks and other informal situations vanished from the online version of the school.

There are *other asymmetries between students and teachers that amplified anomie, conflict and negative emotions in the classroom*. There is only one teacher for many students, with classrooms often enrolling more than 30 pupils. Teachers have formal authority, yet they can be overwhelmed by Students’ diverse aims, tactics, and resources. Any solution fit-for-all to the challenges raised by the sudden digitalization of learning leads to mismatches and discontents. Professors’ authority has also been eroded by their effort to keep up with technology, with pupils often displaying more tech savvy, the need to placate numerous administrative requirements, and the officially mandated relaxation of standards for student assessment.

It is also notable *what is absent from Students’ accounts.* For example, there is no awareness of an increased fragmentation of attention caused by multitasking in the digital space, documented in studies of online learning (Ettinger and Cohen, 2020; Bruineberg and Fabry, 2021; Clinton-Lisell, 2021). Students do not register the abundance of digital stimuli as a loss of an opportunity for focus. While they do appreciate that *less* visibility and supervision is *more* freedom, they do not appreciate that *more* digital stimuli may, in fact, lead to *less* freedom in orienting their attention.

Another notably absent from our data concerns the *hidden benefits brought by opportunistic deviant behaviors for students and teachers*, enabled by the disruptive transition to digital learning. In both groups, there was opportunity for minimizing effort, missing classes, and cutting corners, as documented in journalistic investigations (Miron, 2021). These mutually accepted forms of deviance from formal requirements are not reflected in participants’ own accounts and evaluations of this period.

This first instance of massive digital education was affected by participants’ lack of preparation, with uncertain expectations, and rules that were missing, conflicting or frequently changing. A future recourse to online school will benefit from a better institutionalization and improved equipment and infrastructure but will inherit participants’ fatigue and negative expectations. It is possible that persistent, systematic inequalities will become more pronounced as regards educational experiences and learning outcomes, as all participants have had time to adjust and use the uneven resources at their disposal to better accommodate this disruptive situation.

Overall, our study on online learning and its influence on subjective well-being points toward *ambivalent and heterogeneous influences of online school on well-being*, through generalized overflow effects and through failed or successful identity work and presentation of self. Depending on their individual and collective results in managing this sudden leap in the digital space, both students and teachers experienced various types and degrees of enhancement or degradation of their well-being. “Identity work” (Schwalbe and Mason-Schrock, 1996) and the “presentation of self” (Goffman, 1956) have proven to be useful sensitizing concepts for understanding the controversies related to online learning and, specifically, the use of webcams, and for mapping the Students’ and teachers’ stakes.

## Study Limitations

This article is based on an exploratory, qualitative analysis of an availability-based subsample of students and teachers. Our qualitative approach was appropriate for identifying multiple and conflicting influences of overflow effects, digital identity work, and presentation of self on teachers’ and Students’ well-being. Through a comparative approach, we could highlight both congruent and contrasting perspectives. Although it is quite large, our sample is not representative of the entire Romanian student and teacher population. A quantitative analysis on a representative sample would add valuable information on dominant tendencies, main sources of variability, and the social stratification of well-being. Moreover, our subsample under-represents certain voices and experiences, such as respondents from rural areas, boys and male teachers, and primary school pupils and teachers. Further research should also discuss how the well-being of minority and other disadvantaged groups, including participants from poor households and pupils and teachers in schools from Roma neighborhoods, was affected during online education.

Another limitation of the study is that it does not include a quantitative content analysis of testimonies, as our approach was exploratory and in-depth, aiming to capture the nuances of the three processes of interest. In our future work we plan to use content analysis software, such as Alcete or Iramuteq, to conduct quantitative analysis, to compare relative frequencies of relevant keywords and to establish distinctive, typical profiles of concerns for students and teachers, respectively.

Students’ and teachers’ answers included a considerable number of positive evaluations and intimate stories and feelings. Even so, another limitation of the study might derive from a possible over-representation of the negative and more policy-amenable aspects of online education. Participants’ answers were spontaneous and brief, elicited by a survey with possible policy impact. Therefore, it is plausible that students and teachers focused on criticisms and problems that could be addressed by authorities, rather than experiences with a higher personal relevance.

## Data Availability Statement

The raw data supporting the conclusions of this article will be made available by the authors, without undue reservation.

## Ethics Statement

Ethical review and approval was not required for the study on human participants in accordance with the local legislation and institutional requirements. Written informed consent from the participants’ legal guardian/next of kin was not required to participate in this study in accordance with the national legislation and the institutional requirements.

## Author Contributions

AH and CR designed the study, reviewed the literature, and analyzed the data. RR and DR offered key technical support and analyzed the data. All authors contributed to the article and approved the submitted version.

## Conflict of Interest

The authors declare that the research was conducted in the absence of any commercial or financial relationships that could be construed as a potential conflict of interest.

## Publisher’s Note

All claims expressed in this article are solely those of the authors and do not necessarily represent those of their affiliated organizations, or those of the publisher, the editors and the reviewers. Any product that may be evaluated in this article, or claim that may be made by its manufacturer, is not guaranteed or endorsed by the publisher.

## Footnotes

1 According to the Ministry of Education, the students *“have the obligation to participate in activities carried out through technology and the Internet; otherwise, the student is considered absent and the absence is recorded in the catalog, except for in justified situations”* (Ministry of Education, 2020b). The formal norms do not mention if it is mandatory for students to have the webcam turned on in order to be counted as present in class.

## Annex

### Questionnaire for Teachers

1. To what extent are you comfortable with delivering online classes?
  • To very large extent
  • To large extent
  • To moderate extent
  • To low extent
  • To very low extent
  • No extent
2. How did the quality of education change in 2020–2021 school year compared to the previous one?
  • It has increased
  • It has decreased
  • It has not changed
3. What do you need for increasing the quality of online classes?
  • High-performance personal computer/laptop
  • Adequate working space at home
  • Good internet connection
  • Educational content adapted to online context
  • Motivated students
  • Other
  • Nothing
4. How do you organize the online classes?
  • I organize all lessons according to the timetable
  • I organize part of the lessons using video platforms and for the rest I send individual worksheets
  • I only send individual worksheets for students
  • Other
5. What do you think is the main role you have in the online education system?
  • To facilitate education
  • To deliver information
  • To emotionally support the students
  • Other
6. What methods do you use for teaching in the online educational system?
  • Lectures and exercises
  • Discussions and debates
  • Watching audio-video materials
  • Other
7. To what extent do you succeed in adapting the educational content and teaching approaches to the Students’ needs?
  • To very large extent
  • To large extent
  • To moderate extent
  • To low extent
  • To very low extent
  • No extent
8. To what extent will you continue to use digital methods and tools when schools will reopen for face-to-face activities?
  • To very large extent
  • To large extent
  • To moderate extent
  • To low extent
  • To very low extent
  • No extent
9. Please, briefly describe your experience with online education.
  *The questions were adapted for the students keeping the same meaning of the questions. One new question was added for the students:*
  How would you describe online classes?
  • Interactive
  • Relaxing
  • Boring
  • Useless
  • Other
